# TransplantLines, a biobank and cohort study of solid organ transplant recipients and donors

**DOI:** 10.1007/s10654-025-01258-1

**Published:** 2025-07-02

**Authors:** Anna M. Posthumus, Tim J. Knobbe, Daan Kremer, Antonio W. Gomes-Neto, Isabelle J. C. Dielwart, Jip Jonker, Caecilia S. E. Doorenbos, Michele F. Eisenga, Marco van Londen, Rianne M. Douwes, Lianne M. Nieuwenhuis, Coby Annema, Marieke T. de Boer, Martin H. de Borst, Kevin Damman, Robert A. Pol, C. Tji Gan, Erik A. M. Verschuuren, Hans Blokzijl, Vincent E. de Meijer, Stephan J. L. Bakker, TransplantLines Investigators, Coby Annema, Stefan P. Berger, Hans Blokzijl, Frank A. J. A. Bodewes, Marieke T. de Boer, Kevin Damman, Martin H. de Borst, Arjan Diepstra, Gerard Dijkstra, Rianne M. Douwes, Caecilia S. E. Doorenbos, Michele F. Eisenga, Michiel E. Erasmus, C. Tji Gan, Antonio W. Gomes-Neto, Eelko Hak, Bouke G. Hepkema, Marius C. van den Heuvel, Jip Jonker, Frank Klont, Tim J. Knobbe, Daan Kremer, Coretta van Leer-Buter, Henri G. D. Leuvenink, Marco van Londen, Willem S. Lexmond, Vincent E. de Meijer, Hubert G. M. Niesters, Gertrude J. Nieuwenhuis-Moeke, L. Joost van Pelt, Robert A. Pol, Anna M. Posthumus, Adelita V. Ranchor, Jan Stephan F. Sanders, Marion J. Siebelink, Riemer J. H. J. A. Slart, J. Cas Swarte, Daan J. Touw, Charlotte A. Velde-Keyzer, Erik A. M. Verschuuren, Michel J. Vos, Rinse K. Weersma, Stephan J. L. Bakker

**Affiliations:** 1 Department of Internal Medicine, University Medical Centre Groningen, University of Groningen, Groningen, The Netherlands; 2 Department of Surgery, University Medical Centre Groningen, University of Groningen, Groningen, The Netherlands; 3 Department of Gastroenterology and Hepatology, University Medical Centre Groningen, University of Groningen, Groningen, The Netherlands; 4 Department of Health Sciences, Section of Nursing Science, University Medical Centre Groningen, University of Groningen, Groningen, The Netherlands; 5 Department of Cardiology, University Medical Centre Groningen, University of Groningen, Groningen, The Netherlands; 6 Department of Pulmonary Diseases and Tuberculosis, University Medical Centre Groningen, University of Groningen, Groningen, The Netherlands

**Keywords:** Solid organ transplantation, Solid organ donation, TransplantLines, Cohort profile, Biobank

## Abstract

The TransplantLines Biobank and Cohort Study (NCT03272841) is an ongoing prospective study conducted at the University Medical Centre Groningen, The Netherlands. TransplantLines aims to identify risk factors and biomarkers associated with health problems following solid organ transplantation and donation. Additionally, the study seeks to develop new interventions to reduce symptom burden and improve long-term outcomes, including health-related quality of life, cardiovascular complications, graft failure, and mortality. It includes recipients of (combined) heart, liver, lung, kidney, pancreas, and small bowel transplants, as well as living liver and kidney donors, and deceased (multi-)organ donors. The biobank contains a wide range of biomaterials including whole blood, serum, EDTA-plasma, buffy coat, 24-h urine samples, faeces, hair, nails, and tissues. Data collection includes physical and cognitive assessments, extensive laboratory analysis, metagenomic sequencing, and questionnaires. TransplantLines, initiated in 2015, consists of 5143 participants as of October 2024, among 2312 (45%) females. The mean age was 50 (± 16) years at transplantation, 55 (± 11) years at living donation and 56 (± 15) years at deceased donation. Both cross-sectional and longitudinal biomaterials and data are included. For recipients, longitudinal biomaterials and data were collected at: pre-transplantation, at transplantation, and at 3, 6, 12, 24, and 60 months post-transplantation. For living donors, data were collected at pre-donation, donation, 3 months post-donation, and/or 5 or 10 years post-donation.

**Supplementary Information:**

The online version contains supplementary material available at 10.1007/s10654-025-01258-1.

## Introduction

Solid organ transplantation is the treatment of choice for patients with organ failure. Improvements in immunosuppressive regimens, infection management, perioperative care and surgical techniques have led to tremendously improved early post-transplant outcomes [1]. As life expectancy increases, health-related quality of life (HRQoL) becomes more relevant, especially since many recipients experience a substantial symptom burden that impacts daily life [2–5]. Additionally, graft failure and increased mortality rates continue to shape long-term outcomes in the solid organ transplant population. To address these issues, the TransplantLines Biobank and Cohort study was established [6]. This study aims to identify risk factors and biomarkers associated with health problems after solid organ transplantation and donation, and to identify new interventions to reduce symptom burden and improve long-term outcomes, including cardiovascular complications, graft failure, mortality and patient reported outcomes such as HRQoL and societal participation.

## Methods

### Design of the TransplantLines biobank and cohort study

TransplantLines is an ongoing, single-centre, observational cohort study conducted at the University Medical Centre Groningen (UMCG), The Netherlands. It includes recipients of all types of (combined) solid organ transplants as well as living and deceased organ donors. All (potential) solid organ transplant recipients and (potential) living donors at the UMCG were invited to participate. Both cross-sectional and longitudinal biomaterials and data are included. The comprehensive inclusion of a substantial number of recipients and donors across all types of solid organs, alongside extensive demographic, clinical, physical, cognitive and psychosocial data, as well as extensive laboratory testing, combined with a broad array of biomaterials, such as 24-h urine samples, hair, nails, faeces, and perioperatively collected tissues, next to whole blood, buffy coat, serum and plasma stored in various forms, makes this study unique. There are several studies embedded in TransplantLines:

- Coronary artery calcium (CAC) study (*n* = 203): observational study to assess vascular calcification using a CT-scan in kidney transplant recipients [7].
- Randomized controlled prospEctiVe trial comparing extended-release with immediate-release tacrOlimus; reducing calcineurin inhibitor related toxicity in LUng TransplantatION patients (REVOLUTION) study (n = 149): interventional study to compare Envarsus to Prograft in lung transplant recipients (ClinicalTrials.gov identifier: NCT05001074).
- Vitamin K study (*n* = 39): interventional study to assess the effects of vitamin K supplementation in kidney transplant recipients (EudraCT Number: 2019–004906-88; NTR number: NL7687).
- OPen label multicenter randomized trial comparing standard IMmunosuppression with tacrolimus and mycophenolate mofetil with a low exposure tacrolimus regimen In combination with everolimus in de novo kidney transplantation in Elderly patients (OPTIMIZE) study (*n* = 130): interventional study to examine an alternative immunosuppressive regime for kidney transplant recipients aged ≥ 65 years (ClinicalTrials.gov identifier: NCT03797196).
- SEnsory Neuropathy Scores (SENS) study (*n* = 169): a study assessing sensory neuropathy scores in kidney transplant recipients, validated using quantitative sensory testing and nerve conduction studies (ClinicalTrials.gov identifier: NCT04664426).
- Trajectory of Immunosuppression-Caused Tremor In Kidney Transplant Recipients (TICTIK) study (*n* = 72): this prospective observational study investigates the progression of tremor after kidney transplantation using various tests. Tremor is assessed before transplantation and at different moments after transplantation (ClinicalTrials.gov identifier: NCT06488651).

### Study visits

All laboratory test results recorded in the electronic medical patient files of the participants are included in TransplantLines. Generally, transplant recipients undergo frequent follow-up visits during the first year after transplantation, with the frequency gradually decreasing over time. After the first year, stable kidney transplant recipients are typically followed up at different hospitals, but continue to undergo comprehensive laboratory testing at least once a year at the UMCG. Recipients of other organ transplants generally maintain regular follow-up visits at the UMCG. Living kidney donors receive typically follow up at 3 months, 5 years, and 10 years post-donation.

**Fig. 1:**
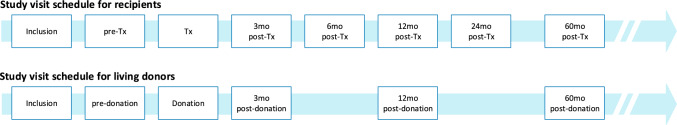
Study visits in the longitudinal cohort of TransplantLines. Abbreviation: Tx: transplantation

Additionally, participants of TransplantLines underwent study visits that included comprehensive laboratory testing, biobanking and for a subset of the participants also a physical or cognitive assessment. Potential recipients and those < 1 year post-transplant at the time of inclusion in TransplantLines were included in the longitudinal cohort. They received study visits at pre-transplantation, at transplantation, and at 3, 6, 12, 24, and 60 months post-transplantation. Recipients who were ≥ 1 year post-transplant at inclusion were included in the cross-sectional cohort, and underwent one single study visit. Preceding the study visits, participants completed self-administered questionnaires at home. In case of a re-transplantation, follow-up for the previous transplantation ended, and a new transplantation trajectory was started in the longitudinal cohort for the existing participant, with the same specified time points for the study visits after the re-transplantation. Additionally, kidney transplant recipients undergoing graft biopsy for suspected rejection underwent an extra study visit with comprehensive laboratory testing and biobanking. Living donors received study visits at pre-donation, donation, 3 months post-donation, and/or 5 or 10 years post-donation, with questionnaires preceding these visits regarding the donation trajectory. An overview of the study visits in the longitudinal cohort is shown in Fig. 1.

### Data collection

#### Clinical and laboratory measurements

For transplant recipients and living donors, demographic and laboratory test results are extracted from their medical records. Transplant- and donor-related data, including the indication for transplantation, donor type, and HLA mismatches, are obtained from the Eurotransplant registry. For deceased donors, data such as demographics, cause of death, donated organs, and medical history are also derived from the Eurotransplant registry. An overview of donor and recipient data is presented in Supplementary Table S1. All laboratory data recorded in the patients’ medical records are included. In addition to routine laboratory measurements for clinical care, extensive testing is conducted during TransplantLines study visits to minimize indication bias (see Table 1).

**Table 1:** Laboratory measurements performed at the study visits of TransplantLines

**Chemistry**	**Hematology**	**Blood gas**
ALT (U/L)	Basophils (count, × 10^9^/L)	Calcium (ionized) (mmol/L)
Albumin (g/L)	Eosinophils (count, × 10^9^/L)	Chloride (mmol/L)
Alkaline phosphatase (U/L)	Erythroblasts (count, × 10^9^/L)	Glucose(mmol/L)
AST (U/L)	Erythrocytes (count, × 10^12^/L)	Lactate (mmol/L)
Bilirubin, direct (µmol/L)	Ferritin (µg/L)	Potassium (mmol/L)
Bilirubin, total (µmol/L)	Folic acid (nmol/L)	Sodium (mmol/L)
Calcium (mmol/L)	Haptoglobin (g/L)	Venous Base Excess (mmol/L)
Chloride (mmol/L)	Hemoglobin (g/dL)	Venous HCO_3_ (mmol/L)
Cholesterol, HDL (mmol/L)	Hematocrit (%)	Venous pCO_2_ (mmol/L)
Cholesterol, LDL (mmol/L)	Immature granulocytes (count, × 10^9^/L)	Venous pH (mmol/L)
Cholesterol, total (mmol/L)	Leukocytes (count, × 10^9^/L)	Venous pO_2_ (mmol/L)
Creatine Kinase-total (U/L)	Lymphocytes (count, × 10^9^/L)	Venous saturation (mmol/L)
C-reactive protein (mg/L)	Mean corpuscular volume (fL)	
Cystatin C (mg/L)	Neutrophils (count, × 10^9^/L)	**Spot urine**
Gamma-Glutamyltransferase (U/L)	Red Cell Distribution Width-cv (%)	Bacteria (count per µL)
Glucose (mmol/L)	Reticulocytes (count, × 10^9^/L and ‰)	Bilirubin (strip, negative/positive)
HS-troponine T (ng/L)	Thrombocytes (count, × 10^9^/L)	Erythrocytes (count per µL)
HbA1c (% and mmol/mol)	Transferrin (g/L)	Glucose (strip, negative/positive)
Iron (µmol/L)	Transferrin saturation (%)	Haem (strip, negative/positive)
Creatinine (µmol/L)	Total Iron Binding Capacity (µmol/L)	Ketones (strip, negative/positive)
Lactate Dehydrogenase (U/L)	Vitamin B12 (pmol/L)	Leukocytes (count per µL)
Magnesium (mmol/L)	Vitamin C (µmol/L)	Leukocytes (strip, negative/positive)
NT-proBNP (ng/L)		Nitrite (strip, negative/positive)
Phosphate (mmol/L)	**Hemoximetry**	Protein (strip, negative/positive)
Potassium (mmol/L)	Hemoglobin (g/dL)	Urobiline (strip, negative/positive)
PTH (ng/L)	Carboxyhemoglobin (%)	
Sodium (mmol/L)	Methemoglobin (%)	**24-h urine**
Total protein (g/L)		Volume (mL/24 h)
Triglycerides (mmol/L)	**Immunosuppressants**	Number of missed portions (count)
Urea (mmol/L)	Tacrolimus (µg/L)	Albumin (mmol/L and mg/24 h)
Uric acid (µmol/L)	Everolimus (µg/L)	Chlorine (mmol/L and mg/24 h)
	Mycophenolic acid (µg/L)	Creatinine (mmol/L and mg/24 h)
	Sirolimus (µg/L)	Magnesium (mmol/L and mg/24 h)
	Ciclosporin (µg/L)	pH
		Phosphate (mmol/L and mg/24 h)
		Potassium (mmol/L and mg/24 h)
		Sodium (mmol/L and mg/24 h)
		Total protein (mmol/L and mg/24 h)
		Urea (mmol/L and mg/24 h)

*NT-proBNP:* n-terminal pro-b-type natriuretic peptide

#### Study visit

**Fig. 2:**
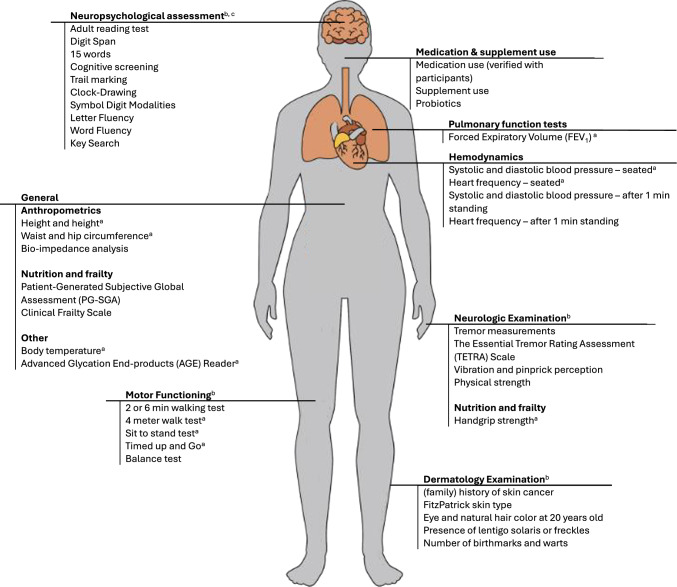
Overview of the assessments conducted during study visits of TransplantLines. ^a^: measured multiple times. ^b^: participants were randomized to either a physical assessment (motor function, neurological and dermatology examination) or a cognitive assessment (neuropsychological assessment). ^c^: collected until March 2021

Study visits were conducted at the outpatient clinic following a standard operating procedure (SOP) and performed by trained researchers. During each study visit, data were collected on blood pressure, heart rate, temperature, anthropometry, body composition (measured by bioimpedance analysis), muscle strength, lung function, nutritional status, frailty status, advanced glycation end products (AGEs), and medication use, among others. Additionally, participants either underwent additional physical or cognitive-focused assessments. The additional physical assessment included tests for standing balance, 2- or 6-min walking test, 4-m walk, five-times-stand, timed-up-and-go, and the 9-hole peg test, as well as a neurological examination focused on polyneuropathy and tremor. The additional cognitive-focused assessment included a neuropsychological assessment measuring memory, mental speed, attention and executive functioning. Cross-sectional and 12-month post-transplant study visits also included a dermatological assessment. After 2021, physical study visits were discontinued, and data on blood pressure, heart rate, anthropometry, and medication use were extracted from medical records. An overview of the assessments is presented in Fig. 2.

#### Questionnaires

Participants filled out questionnaires prior to each study visit (except for the study visits at transplantation and at 3 months post-transplant). These questionnaires evaluated physical and mental health symptoms, including fatigue and sleep quality. Lifestyle factors such as smoking behaviour, alcohol use, and physical activity were also assessed. Psychological items received significant attention, with measures for coping strategies, cognitive functioning, well-being, and symptoms of anxiety and depression. The questionnaires also addressed social functioning, including work-related aspects like employment and work performance, societal participation, family relationships, and social support. Therapy adherence was evaluated as well. Furthermore, dietary intake was estimated using food frequency questionnaires. An overview of the questionnaires is provided in Table 2.

**Table 2:** Overview of questionnaires

Questionnaire	Key measurement area	Recipients	Donors
*General*			
Demographics and Socioeconomic Status Questionnaire	Demographics and Socioeconomic Status	X	X
Smoking behaviour	Smoking behaviour	X	X
Food Frequency Questionnaire (FFQ)	Food intake including alcohol intake	X	X
Alcohol Use Disorders Identification Test (AUDIT)	Alcohol use disorders	X	X
EuroQol 6 Dimensions (3-level version) (EQ6D-3L)	Health-related quality of life	X	X
RAND-36 version 1/ Short Form-12 Health Survey (SF12)^b^	Health-related quality of life	X	X
Checklist Individual Strength 20/8 Revised (CIS20R or CIS8R)^c^	CIS20R: fatigue severity (subjective experience of fatigue), concentration problems, reduced motivation and reduced activity level CIS8R: fatigue severity	X	X
Modified Transplant Symptom Occurrence and Symptom Distress Scale 59 revised (MTSOSDS-59R)	Side-effects of Immunosuppressive medication	X	X
Pittsburgh Sleep Quality Index (PSQI)	Sleep quality	X	X
Gastrointestinal health	Gastrointestinal function and complaints	X	X
Bristol Stool Chart	Stool consistency assessment	X	X
Basel Assessment of Adherence to Immunosuppressive Medication Scale (BAASIS)	Medication adherence	X	X
Carolinas Comfort Scale	Pain		X
Pain after donation	Pain		X
Post-operative pain	Pain		X
Reflection on donation	Reflection on the donation experience		X
*Physical functioning*			
Short Questionnaire to Assess Health-enhancing Physical Activity (SQUASH)^a^	Physical activity	X	X
Dutch Standard for Healthy Physical Activity (NNGB)	Physical activity	X	X
Insufficient Physical Activity Behaviour in the Netherlands (OBiN)	Sedentary behaviour	X	X
*Psychological functioning*			
World Health Organization Well-Being Index (WHO-5)	Well-being	X	X
State-Trait Anxiety Inventory (6-item version) (STAI6)	Anxiety symptoms	X	X
Patient Health Questionnaire (9-item version) (PHQ9)	Depressive symptoms	X	X
Pearlin Mastery Scale	Personal control	X	X
Utrecht Coping List (47-item version) (UCL-47)	Coping	X	X
Cognitive Failure Questionnaire (CFQ)	Subjective cognitive functioning	X	X
Transplant Effects Questionnaire (TxEQ)^d^	Emotional response to the receipt of a transplanted organ	X	
Donation decision Regret Scale	Regret about the donation decision		X
*Social functioning*			
Utrecht Scale for Evaluation of Rehabilitation-Participation (USER-P)	Societal participation	X	X
Work and work ability	Work and work ability	X	X
Work Role Functioning Questionnaire (WRFQ)^a^	Work functioning	X	X
Family Assessment Device (General Functioning) (FAD-GF)	Family dynamics	X	X
Active Engagement, Protective Buffering and Overprotection (Social Functioning Scale) (ABO)^a^	Support from partner	X	
Social Support List-Interaction (SSL-I)	Social support	X	X

^a^Questionnaire administered until 2021

^b^The RAND-36 was temporarily replaced by the SF-12

^c^The CIS20R was replaced by the CIS8R in 2021

^d^after 2021 only administered 6 months after transplantation

#### Biomaterials

At each study visit, a wide range of biomaterials is collected, including various blood-derived components such as buffy coat, plasma (citrate, EDTA, and heparin), PAXgene® (blood RNA tube – QIAGEN), serum, and whole blood. Additionally, 24-h urine collections (both non-acidified and acidified), faeces, hair, and nails are collected. At the time of a kidney allograft biopsy, additional blood and urine samples are collected and immediately stored on ice. During kidney transplantations, additional samples are collected from the skin, subcutaneous tissue, perivascular fat, ureter, external iliac artery, renal artery, perirenal fat, and kidney biopsies. Since 2021, the collection of blood stored in citrate, heparin, and PAXgene® (blood RNA tube – QIAGEN), as well as acidified 24-h urine, faeces, hair and nails, has been discontinued. All samples are stored at -80 °C. Additionally, tissue samples from the kidney graft and surrounding tissues, collected for clinical care, are available for research purposes.

## Results

### Population characteristics

TransplantLines, initiated in 2015, is an ongoing study of in total 5143 participants as of October 2024, among which 3397 (potential) solid organ transplant recipients and 1746 (potential) donors. The participation rate was 80%.

The 5143 participants consist of 1712 kidney, 602 liver, 390 lung, 144 heart, 1 pancreas and 6 small bowel transplant recipients, as well as 145 transplant recipients who underwent sequential and/or combined multi-organ transplantations (excluding re-transplantations), and another 397 patients assessed for potential solid organ transplantation (who were either (1) considered ineligible and removed from the waitlist, (2) deceased, or (3) still on the waiting list for a transplant). Additionally, 994 living kidney donors, 9 living liver donors, 3 living kidney-liver donors, as well as 341 potential donors assessed for living kidney donation, and 399 deceased donors were included (see Table 3). Among the 5143 participants, 2312 (45%) were female, with a mean age of 50 (± 16) years at transplantation, 55 (± 11) years at living donation, and 56 (± 15) years at deceased donation.

**Table 3:** Characteristics

	TransplantLines *n* = 5143
*Age, years*	
At time of first transplantation	50 (± 16)
At time of donation	
Living	55 (± 11)
Deceased	56 (± 15)
*Sex, n (%)*	
Male	2831 (55)
Female	2312 (45)
*Transplantation type, n (%)*	
Screening^a^	
Kidney	137 (4)
Liver	131 (4)
Lung	81 (2)
Heart	38 (1)
Small bowel	8 (< 1)
Liver-Lung	1 (< 1)
Heart–Lung	1 (< 1)
Kidney	1712 (50)
Liver	602 (18)
Lung	390 (11)
Heart	144 (4)
Pancreas	1 (< 1)
Small bowel	6 (< 1)
Sequential and/or combined multi-organ transplantations	
Kidney-pancreas	52 (2)
Kidney-liver	44 (1)
Kidney-lung	17 (1)
Heart–lung	9 (< 1)
Other^b, c^	23 (1)
*Donation type, n (%)*	
Screening^a, d^	341 (19)
Kidney	994 (57)
Liver	9 (1)
Liver and kidney	3 (< 1)
Deceased	
DBD	129 (7)
DCD	270 (15)
*Available of participants, n (%)*^*e*^	
Biomaterials	4829 (94)
Questionnaires	3988 (78)

*DBD*: donation after brain death, *DCD*: donation after circulatory death

^a^are not (yet) transplanted or did not (yet) donate

^b^other sequential and/or combined multi-organ transplantations, which include kidney-heart, kidney-small bowel, kidney-liver-lung, kidney-lung-pancreas, heart-liver, liver-lung, liver-pancreas and liver-pancreas-small bowel transplantations

^c^including one kidney transplant recipients who previously donated a kidney

^d^only kidney donors included, no data available of liver donors

^e^available for at least one moment per participant

**Fig. 3:**
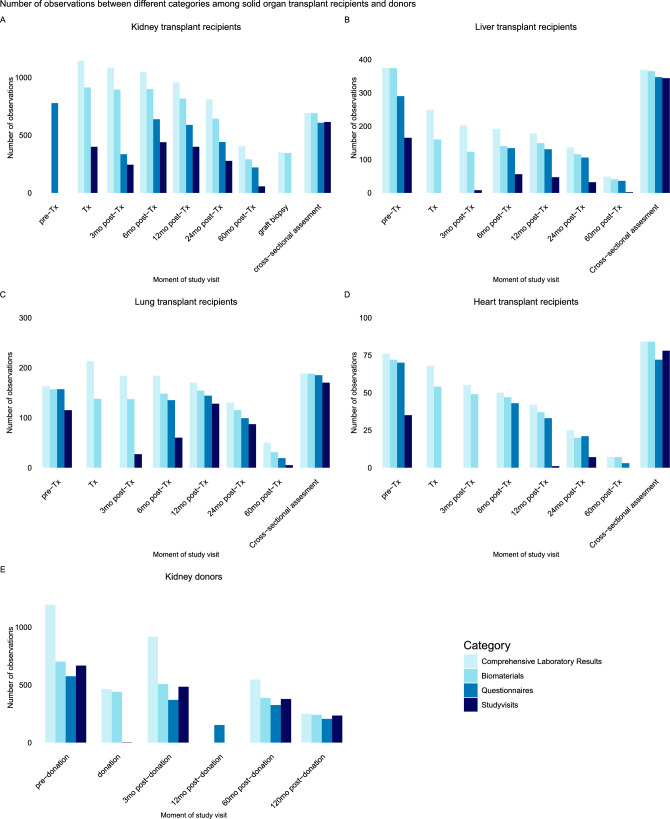
Overview of the number of observations per patient group at TransplantLines study visits. This figure shows available data or materials per category: comprehensive laboratory testing, biomaterial collection, questionnaire data, and completed study visits. A: kidney transplant recipients, B: liver transplant recipients, C: lung transplant recipients, D: heart transplant recipients, E: kidney donors

An overview of the data collected in the TransplantLines cohort is provided in Fig. 3 and Supplementary Figure S1.

## Discussion

The TransplantLines Biobank and Cohort study is, to our knowledge, one of the largest studies including all types of organ transplant recipients and donors. The availability and combination of various types of biomaterials, along with extensive clinical, laboratory, and psychological data, make the study unique. This combination of data allows for domain-specific and inter- and multidisciplinary analyses.

### Key findings and publications

TransplantLines data have been used in over 88 published studies, with many more ongoing research projects. Most research has focused on identifying risk factors after transplantation, which is one of the key reasons TransplantLines was established.

Research has demonstrated that the gut microbiome in solid organ transplant recipients differs significantly from that of healthy controls, with many recipients experiencing gut microbiome dysbiosis. This dysbiosis has been linked to reduced long-term survival and worse health-related quality of life [8–11]. Other research has focused on patient-reported outcomes in kidney transplant recipients, which include studies into the trajectory and identification of factors such as hand dexterity, iron deficiency and proton-pump inhibitor use, and symptoms such as sleep quality, tremor and fatigue that may affect health-related quality of life [2, 4, 5, 12]. Additionally, societal participation and work functioning have been assessed [3, 13]. Furthermore, analysis of the neuropsychological assessment data revealed that many kidney transplant recipients experience mild cognitive impairment which may impair health-related quality of life [14].

Other studies have focused on developing new techniques or optimizing existing ones. In the field of metabolomics, research has focused on optimizing and developing new techniques for small-molecule profiling and data normalization [15], and has shown that untargeted SWATH mass spectrometry-based metabolomics can help identify and confirm medication exposure in patients [16]. Another example is the quantification of plasma tacrolimus levels. Traditionally, tacrolimus is measured in whole blood samples; however, this approach is suboptimal, as erythrocyte-bound tacrolimus does not accurately represent the active fraction. A dedicated research line explores the potential of plasma-based tacrolimus measurement [17].

Biomarker research in blood and urine predicting post-transplant outcomes is an important focus of TransplantLines. For example, the potential of the biomarkers plasma endotrophin, glycA, citrate, symmetric dimethylarginine, fibroblast growth factor 23 and trimethylamine-N-oxide has been assessed [18–24].

In addition to biomarkers, TransplantLines has identified potential contributors to adverse outcomes after transplantation. For example, a study examined the potential effect of heavy metals and found that concentrations of lead are associated with kidney graft failure [25]. Other areas of research using TransplantLines data include nutrition and nutritional status [26, 27] and pharmacology [28, 29], among others.

In living kidney donors, TransplantLines data showed that measurement of cystatin C is of use when predicting post-donation kidney function [30]. Besides, it seems that BMI is not associated with peri- and postoperative complications [31]. However, a higher BMI, waist circumference and waist-to height ratio is associated with a lower glomerular filtration rate [32].

The vitamin K study, assessing the effects of vitamin K supplementation among kidney transplant recipients, showed that vitamin K supplementation may prevent progression of arterial stiffness and therefore may have vascular effects [33]. The CAC-study assessing coronary artery calcification at 12 months after kidney transplantation identified several potential determinants of this calcification [7].  The other studies that are embedded in TransplantLines are ongoing.

### Strengths and weaknesses

The main strengths of the TransplantLines study lie in its large, longitudinal design; the inclusion of all types of solid organ transplant recipients as well as both living and deceased donors; and the availability and combination of various biomaterials combined with extensive clinical, laboratory, and psychological data. Another key strength is the representativeness of the cohort, given the participation rate of 80%. Due to its observational design, the study allows for the generation of numerous hypotheses, providing valuable insights into clinical, social, psychological, and biochemical factors. These insights can serve as the foundation for follow-up or interventional studies. A limitation of TransplantLines is that it is a single-centre study conducted in a tertiary setting. As a result, some data may be lost due to follow-up in primary and secondary care.

### Collaboration

TransplantLines is open to collaborations with other researchers. To access the data, researchers can obtain and submit a data request form to datarequest.transplantlines@umcg.nl. The TransplantLines scientific research committee reviews all submitted requests on a monthly basis, evaluating their rationale and feasibility. If approved, the requested data will be provided after the necessary data transfer agreements between institutions have been completed, where applicable.

## Supplementary Information

Below is the link to the electronic supplementary material.

Supplementary file1 (DOCX 319 kb)

## Declarations

### TransplantLines Investigators

Coby Annema, Stefan P Berger, Hans Blokzijl, Frank AJA Bodewes, Marieke T de Boer, Kevin Damman, Martin H de Borst, Arjan Diepstra, Gerard Dijkstra, Rianne M Douwes, Caecilia SE Doorenbos, Michele F Eisenga, Michiel E Erasmus, C Tji Gan, Antonio W Gomes-Neto, Eelko Hak, Bouke G Hepkema, Marius C van den Heuvel, Jip Jonker, Frank Klont, Tim J Knobbe, Daan Kremer, Coretta van Leer-Buter, Henri GD Leuvenink, Marco van Londen, Willem S Lexmond, Vincent E de Meijer, Hubert GM Niesters, Gertrude J Nieuwenhuis-Moeke, L Joost van Pelt, Robert A Pol, Anna M Posthumus, Adelita V Ranchor, Jan Stephan F Sanders, Marion J Siebelink, Riemer JHJA Slart, J Cas Swarte, Daan J Touw, Charlotte A te Velde-Keyzer, Erik AM Verschuuren, Michel J Vos, Rinse K Weersma, Stephan JL Bakker.
