# Supplementary material for: TransplantLines, a biobank and cohort study of solid organ transplant recipients and donors
Source: Eur J Epidemiol. 2025 Jul 2;40(8):969–79. doi: 10.1007/s10654-025-01258-1 (PMC12374880; doi:10.1007/s10654-025-01258-1)
Supplement: Supplementary file 1 — Supplementary file1 (DOCX 319 kb) [file 10654_2025_1258_MOESM1_ESM.docx]

Journal: European Journal of Epidemiology

**Cohort Profile: TransplantLines, a Biobank and Cohort Study of Solid Organ Transplant Recipients and Donors**

Anna M. Posthumus^1^*****; Tim J. Knobbe^1^*****; Daan Kremer^1^; Antonio W. Gomes-Neto^1^; Isabelle J.C. Dielwart^1,2,^; Jip Jonker^1^; Caecilia S.E. Doorenbos^1^; Michele F. Eisenga^1^; Marco van Londen^1^; Rianne M. Douwes^3^; Lianne M. Nieuwenhuis^2^; Coby Annema^4^; Marieke T. de Boer^2^; Martin H. de Borst^1^; Kevin Damman ^5^; Robert A. Pol^2^; C. Tji Gan^6^; Erik A.M. Verschuuren^6^; Hans Blokzijl^3^; Vincent E. de Meijer^2^**^#^**; Stephan J.L. Bakker^1^**^#^**

ON BEHALF OF TransplantLines Investigators

*contributed equally as first author

^#^contributed equally as senior author

^1^: University of Groningen, Department of Internal Medicine, University Medical Centre Groningen, Groningen, The Netherlands.

^2^: University of Groningen, Department of Surgery, University Medical Centre Groningen, Groningen, The Netherlands.

^3^: University of Groningen, Department of Gastroenterology and Hepatology, University Medical Centre Groningen, Groningen, the Netherlands

^4^: University of Groningen, Department of Health Sciences, Section of Nursing Science, University Medical Centre Groningen, Groningen, The Netherlands.

^5^:University of Groningen, Department of Cardiology, University Medical Centre Groningen, Groningen, The Netherlands.

^6^: University of Groningen, Department of Pulmonary Diseases and Tuberculosis, University Medical Centre Groningen, The Netherlands.

**Correspondence**: Stephan J.L. Bakker. Division of Nephrology Department of Internal Medicine, University Medical Centre Groningen, Hanzeplein 1, P.O. Box 30.001, 9713 GZ Groningen, The Netherlands. Tel: +310503613677. E-mail: [s.j.l.bakker@umcg.nl](mailto:s.j.l.bakker@umcg.nl).

**SUPPLEMENTAL MATERIAL**

**Supplemental Table S1.** Overview of the donor and recipient data in TransplantLines

| **Recipients** | **Deceased Donors** |
| --- | --- |
| Age at transplantation | Age at donation |
| Sex | Sex |
| Primary disease | Cause of death |
| Transplantation date | Donation date |
| Transplantation type | Donor type (DCD/DBD) |
| Sequential and/or combined multi-organ transplantation | Organ(s) donated |
| Dialysis technique^a^ | Length of hospital stay until donation |
| HLA typing and mismatches | History of hypertension |
| Delayed graft function^a^ | History of diabetes |
| Rejection (date, pathology, type, treatment) | History of malignancy |
| Date of graft failure | Full medical history |
| Reason of graft failure | Home medication |
| Current medication | Creatinine at admission |
| Donor type (living/DCD/DBD) | Last creatinine before donation |
| Warm ischemic time 1 | Blood gas analysis |
| Cold ischemic time |  |
| Warm ischemic time 2 | **Living donors** |
| Donor age | Age at screening or donation |
| Donor sex | Sex |
| Donor height and weight | Donation date |
| Donor blood group | Organ(s) donated |
| Donor history of hypertension | Full medical history |
| Donor history of diabetes | Current medication |
| Donor smoking status | Comprehensive laboratory assessment |
|  | Measured GFR pre and post-donation |

^a^: only applicable for kidney transplant recipients. Abbreviations: DBD, donation after brain death; DCD, donation after circulatory death; GFR, glomerular filtration rate.


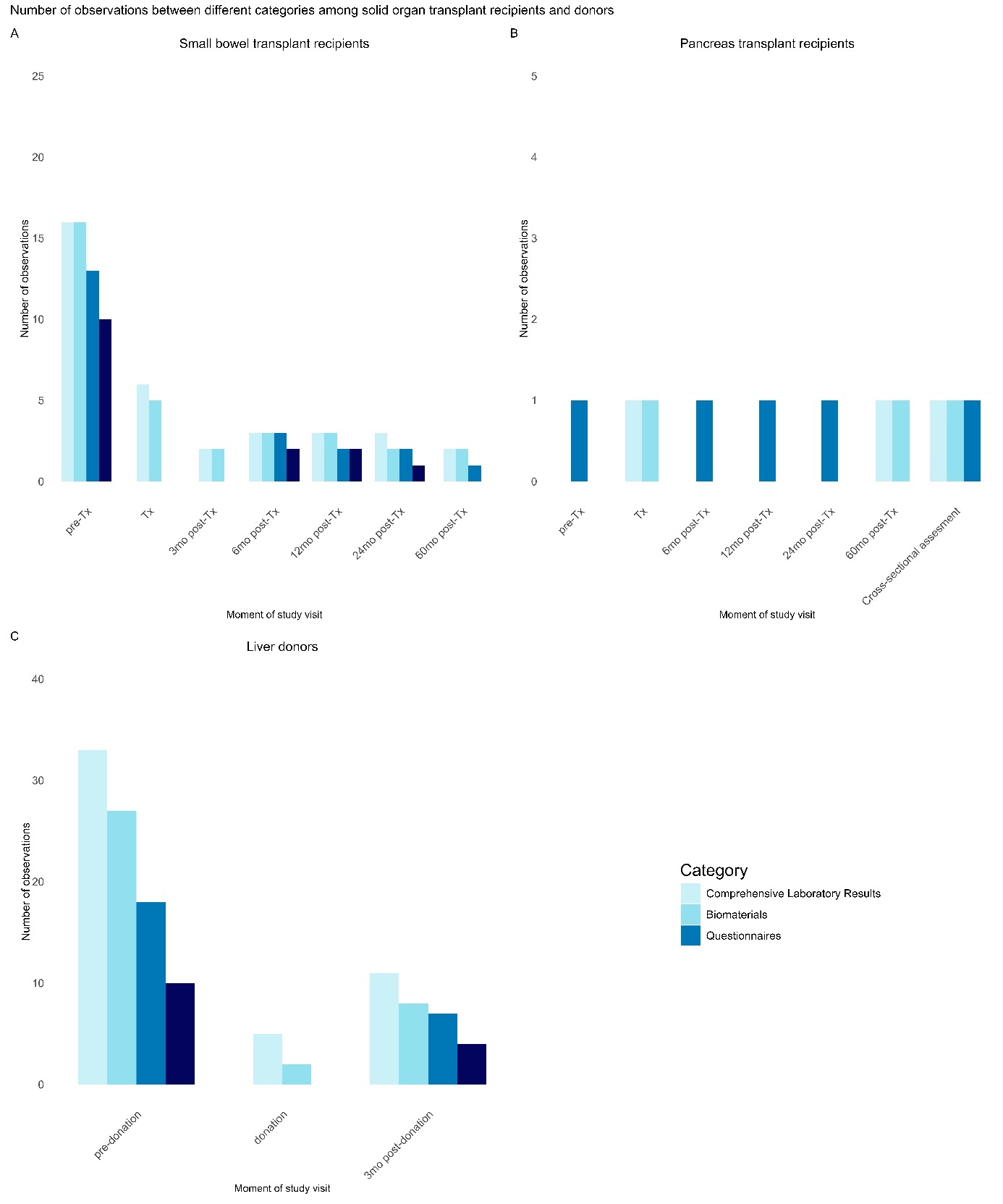


**Supplemental Figure S1: Overview of the number of observations per group of patients at TransplantLines study visits**

This figure shows available data or materials per category - comprehensive laboratory testing, biomaterial collection, questionnaire data, and completed study visits. A: bowel transplant recipients, B: pancreas transplant recipients, C: liver donors.
